# Preoperative exercise therapy for gastrointestinal cancer patients: a systematic review

**DOI:** 10.1186/s13643-018-0771-0

**Published:** 2018-07-24

**Authors:** Sarah A. Vermillion, Alston James, Robert D. Dorrell, Peter Brubaker, Shannon L. Mihalko, Adrienne R. Hill, Clancy J. Clark

**Affiliations:** 1 Bowman Gray Center for Medical Education, Wake Forest University School of Medicine, 475 Vine Street, Winston-Salem, NC 27101 USA; 2 Health and Exercise Science, Wake Forest University, Worrell Professional Center 2164B, PO BOX 7868, Winston-Salem, NC 27109 USA; 3 Department of Physical Medicine and Rehabilitation, Wake Forest Baptist Health, Medical Center Blvd, Winston-Salem, NC 27157 USA; 4 Division of Surgical Oncology, Department of General Surgery, Wake Forest Baptist Health, Medical Center Blvd, Winston-Salem, NC 27157 USA

**Keywords:** Preoperative, Cancer, Surgery, Exercise therapy

## Abstract

**Background:**

Gastrointestinal cancer patients are susceptible to significant postoperative morbidity. The aim of this systematic review was to examine the effects of preoperative exercise therapy (PET) on patients undergoing surgery for GI malignancies.

**Methods:**

In accordance with PRISMA statement, all prospective clinical trials of PET for patients diagnosed with GI cancer were identified by searching MEDLINE, Embase, Cochrane Library, ProQuest, PROSPERO, and DARE (March 8, 2017). The characteristics and outcomes of each study were extracted and reviewed. Risk of bias was evaluated using the Cochrane risk of bias tool by two independent reviewers.

**Results:**

Nine studies (534 total patients) were included in the systematic review. All interventions involved aerobic training but varied in terms of frequency, duration, and intensity. PET was effective in reducing heart rate, as well as increasing oxygen consumption and peak power output. The postoperative course was also improved, as PET was associated with more rapid recovery to baseline functional capacity after surgery.

**Conclusions:**

PET for surgical patients with gastrointestinal malignancies may improve physical fitness and aid in postoperative recovery.

## Background

Gastrointestinal cancer is more common in frail, older adults. With advanced age and deconditioning, patients are more susceptible to postoperative morbidity and mortality [1, 2]. After adjusting for comorbidities and age, frail patients undergoing major abdominal operations, such as pancreaticoduodenectomy, have significantly higher incidence of both minor and major complications as well as increased risk of 30-day mortality [3, 4]. While factors associated with frailty, such as sarcopenia, malnutrition, and poor performance status, are potentially modifiable, the optimal preoperative intervention to alter these factors has not yet been established for gastrointestinal cancers.

Targeted interventions including smoking cessation [5], diabetes management [6], protein supplementation [7], and pulmonary rehabilitation [8] have been developed and introduced into preoperative planning. Using findings from these focused interventions, multi-modality strategies to improve perioperative outcomes are emerging and are best exemplified by the rapid adoption of enhanced recovery pathways in gastrointestinal surgery [9–14]. However, the appropriate components of a preoperative optimization strategy are still under investigation.

Preoperative exercise therapy (PET) has been proposed as one strategy to improve patient performance status, treat sarcopenia, and address disease-associated deconditioning [15–17]. Proposed exercise therapies have varied greatly from focused procedure-specific interventions, such as pelvic floor strengthening for prostatectomy, to whole-body exercise therapy [18–20]. Optimal structure and duration of preoperative exercise therapy may not be generalized across all disease processes. For example, gastrointestinal cancer patients frequently do not have substantial lead time before operation.

In the present study, a systemic review was performed of all prospective clinical trials of PET focused on patients diagnosed with gastrointestinal malignancies. The aim of the current study was to evaluate the clinical benefit of PET on postoperative outcomes in order to identify the optimal strategy to decrease the perioperative risks associated with frailty frequently seen in gastrointestinal cancer patients.

## Methods

Systematic review is reported in accordance with the Preferred Reporting Items for Systematic review and Meta-Analysis (PRISMA) statement [21]. The study protocol was established prior to analysis by study team members. During preliminary analysis, significant study heterogeneity was identified based on study cohort clinico-demographic characteristics, study design, individual study variables, and study endpoints. Therefore, meta-analysis was not performed. This systematic review was considered exempt by the Institutional Review Board at Wake Forest Baptist Health. The current study is not registered in Prospective Register of Systematic Reviews (PROSPERO), and therefore, the study protocol is outlined as follows.

### Eligibility criteria

Eligible studies included prospective clinical trials of preoperative exercise intervention in a study population of adult gastrointestinal cancer patients. While the frail and elderly are most likely to benefit from preoperative exercise, the study included all adults (18 years and older) in the analysis to ensure comprehensive review. Study population comparison was required with or without randomization. We specifically excluded papers with interventions that were limited only to inspiratory muscle training (to prevent postop pulmonary complications) or pelvic floor muscle training (to hasten postop continence following prostatectomy) because we felt that these studies were fundamentally different from general physical exercise. We also excluded any studies without a control group and any single-arm studies. Non-English language studies, unpublished data, or abstracts were excluded from the review.

### Search strategy

Comprehensive list of search terms was developed using a preliminary search by authors CJC and AJ. Review of reference and prior review articles were evaluated, and consultation among content experts helped establish final search strategy. Relevant keyword search used a combination of the following: exercise therapy, surgery, cardiac, elderly, preoperative, joint surgery, abdominal surgery, and outcomes. Studies were identified by searching electronic databases including MEDLINE (1946 to 2017), Embase (1947 to 2017), Cochrane Library, ProQuest, PROSPERO, and Database of Abstracts of Reviews of Effectiveness (DARE). References from each identified article were reviewed. Clinical trial registries were also reviewed including Clinicaltrials.gov, Cochrane Central Register of Controlled Trials (CENTRAL), and World Health Organization International Clinical Trials Registry Platform (ICTRP).

The last database search was performed on March 8, 2017. Screening of search results and study selection was performed independently in an unblinded standardized manner by two reviewers (CJC and AJ). Reviewers were aware of manuscript authorship, institution, and journal. Disagreements between reviewers were resolved by consensus. Initial review of search results included evaluation of study title and abstract. The full text of candidate study articles was then reviewed. Mendeley was used to manage citations and manuscripts (https://www.mendeley.com/).

Information was extracted from each study using a standard data extraction sheet. Data collected included authors, type of institution, year of study, country, subject age, cancer type, operation, type of preoperative intervention, enrollment criteria, physical assessment instruments, and primary and secondary study outcomes. Risk of bias for selected studies was evaluated using the Cochrane risk of bias tool by two independent reviewers (CJC and AJ) [22]. Study reviewers assessed each manuscript for selection, performance, detection, attrition, and reporting biases.

### Statistical analysis

Meta-analysis was not utilized, due to the differences in study design and lack of comparable outcome variables. Therefore, a critical review and analysis of study exercise interventions, inclusion/exclusion criteria, and outcomes was performed. Outcome measures reviewed include typical physiologic measures for exercise intervention (heart rate, peak power output, and pVO_2_), postoperative outcomes (length of stay, complications), and quality of life. With study heterogeneity, additional outcome measures were not able to be evaluated.

## Results

**Fig. 1:**
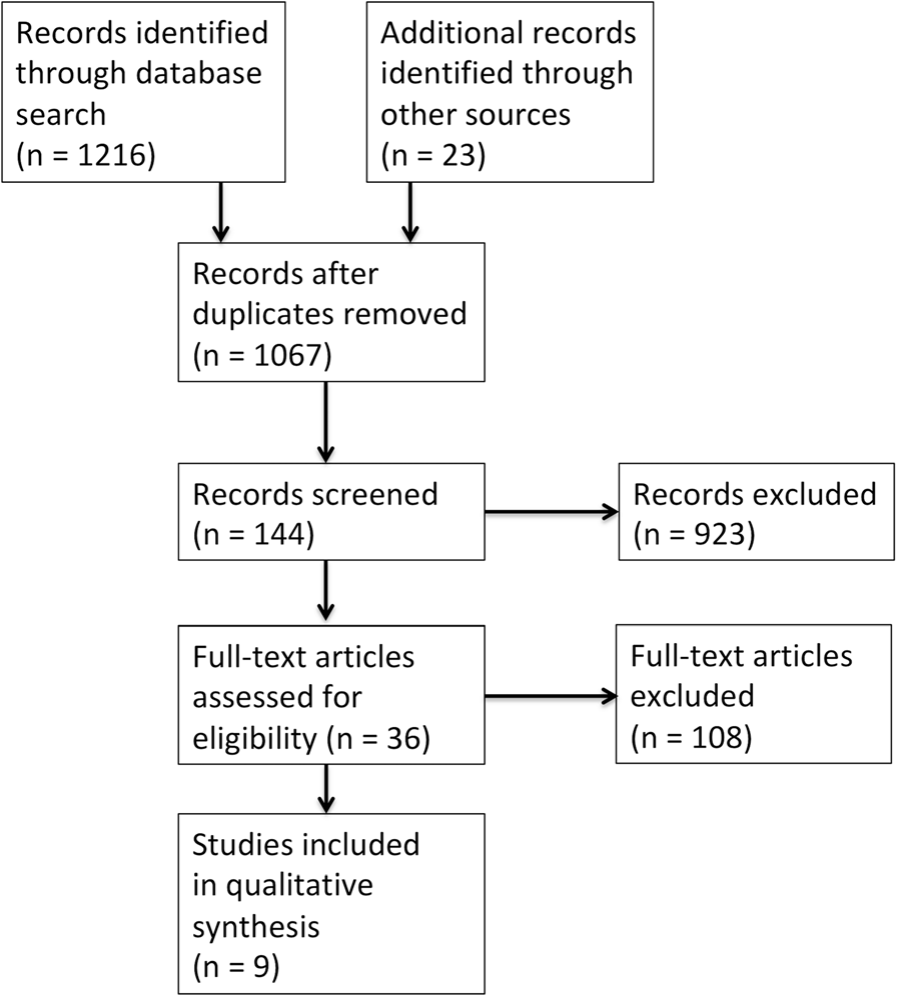
Study selection flow diagram

The search strategy detailed in Fig. 1 identified 1239 results, including 172 duplicate studies. After screening these search results based on title and abstract, 923 studies were determined to be irrelevant and were excluded. The full texts of the remaining 144 studies were reviewed, and studies that contained a single-arm design, non-English language, and unpublished data or consisted of only an abstract were excluded. Thirty-six studies of preoperative exercise intervention were identified. The systemic review was then further narrowed to nine studies that evaluated patients with gastrointestinal cancer [23–31].

### Risk of bias

**Fig. 2:**
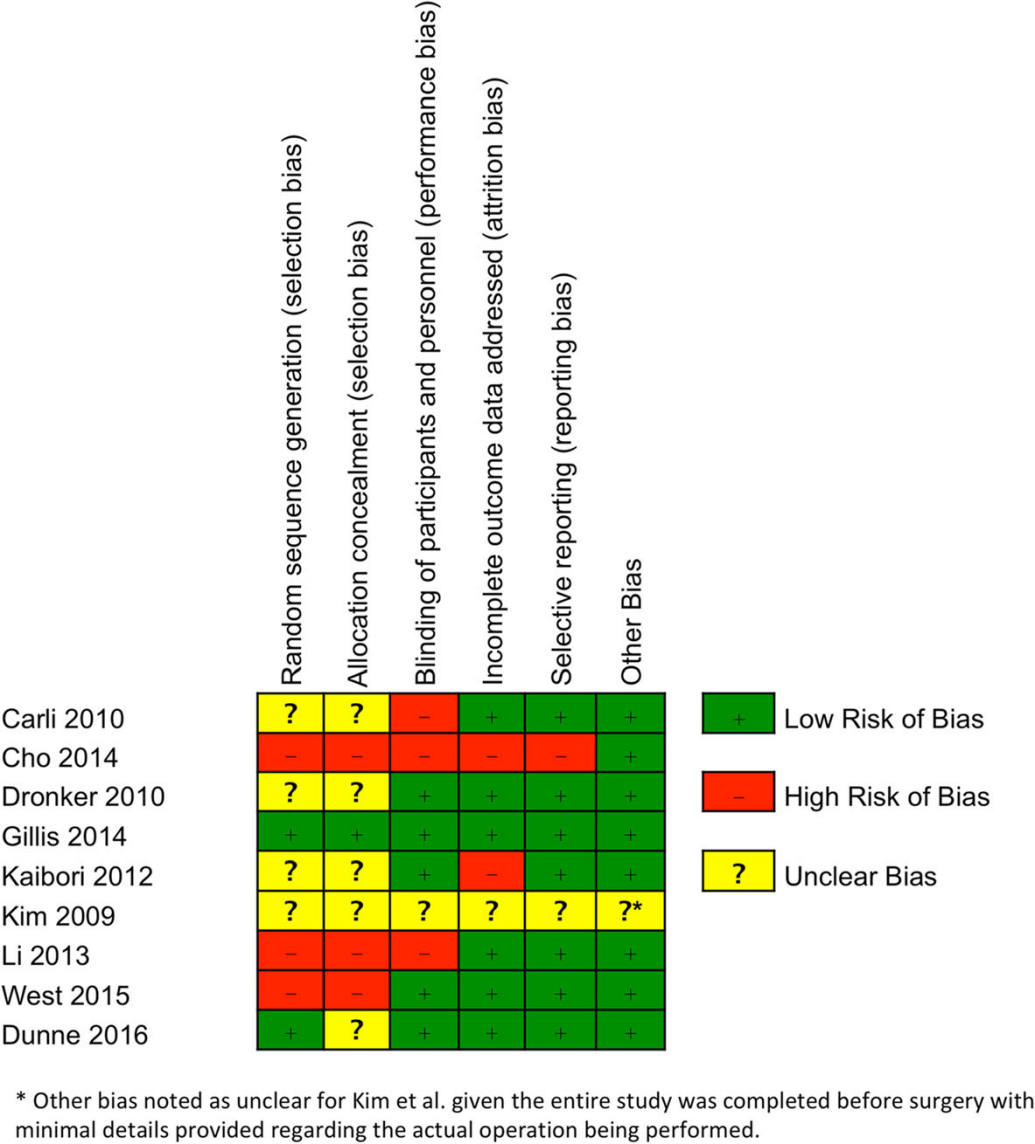
Risk of bias summary based on Cochrane Risk of Bias Tool [22]

**Fig. 3:**
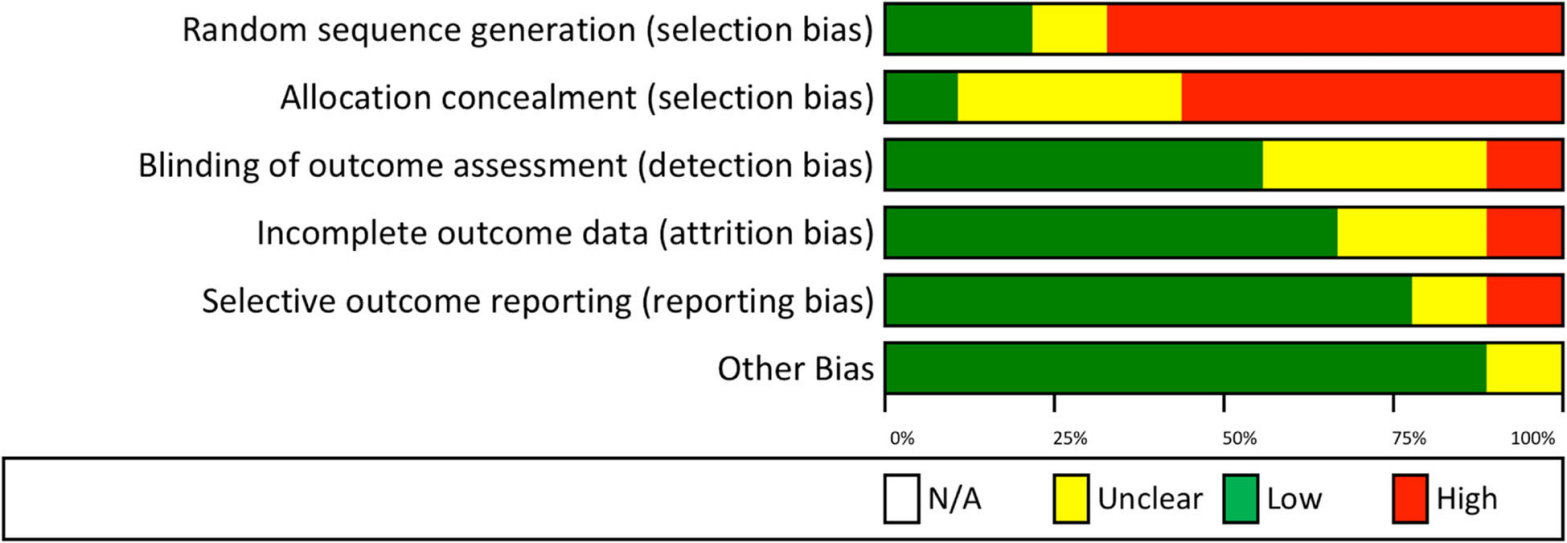
Risk of bias graph based on Cochrane Risk of Bias Tool [22]

Bias within studies was assessed independently by two reviewers, and the results are shown in Figs. 2 and 3. Based on the Cochrane risk of bias tool, five studies demonstrated a high risk of bias [22–24, 28, 30, 31]. Two studies showed an unclear risk of bias [25, 29]. Two studies were determined to have a low risk of bias [26, 27].

### Study characteristics

Of the nine studies that met the eligibility requirements, six were randomized controlled trials [23, 25–29]. Three studies were prospective non-randomized controlled trials [24, 30, 31]. Studies were conducted in Canada (*n* = 4), Japan (*n* = 2), the UK (*n* = 2), and the Netherlands (*n* = 1) [23–31].

### Participants

In total, 534 participants were enrolled in the nine studies reviewed. The number of participants per study ranged from 21 to 112. Patient characteristics are summarized in Table 1. Only one study reported significant differences at baseline between the control and intervention groups [31]. Specifically, West et al. reported significant differences in age, American Society of Anesthesia (ASA) physical classification status, performance status, and predicted mortality [31].

**Table 1:** Summary of patient characteristics

Study	Patient population	Groups	*N*	Age (year)	% male	ASA > II	BMI (kg/m^2^)
Carli et al.	Benign or malignant colorectal cancer	Intervention	58	61 ± 16	59	22%	28 ± 6
		Control	54	60 ± 15	57	20%	27 ± 5
Cho et al.	Gastric cancer	Intervention	18	63.1 (51–76)*	100	NR	26.7 (23.1–31.2)*
		Control	54	66.1 (39–81)*	94.4	NR	25.7 (20.8–34.1)*
Dronkers et al.	Colon cancer	Intervention	22	71.1 ± 6.3	68.2	NR	26.6 ± 3.6
		Control	20	68.8 ± 6.4	80	NR	25.6 ± 3.1
Gillis et al.	Colorectal cancer	Intervention	38	65.7 ± 13.6	55	26%	26.9 ± 4.6
		Control	39	66.0 ± 9.1	69	23%	28.5 ± 4.3
Kaibori et al.	Hepatocellular carcinoma	Intervention	25	68 ± 9.1	68	NR	NR
		Control	26	71.3 ± 8.8	73	NR	NR
Kim et al.	Benign or malignant colorectal cancer	Intervention	14	55 ± 15	64	NR	26.6 ± 5.9
		Control	7	65 ± 9	57	NR	25.3 ± 2.7
Li et al.	Colorectal cancer	Intervention	42	67.4 ± 11	54	19%	27.5 ± 4
		Control	45	66.4 ± 12	64	22%	26.9 ± 6
West et al.	Rectal cancer treated with NACRT	Intervention	22	64 (45–82)*	64	9%	NR
		Control	13	72 (62–84)	69	15%	NR
Dunne et al.	Colorectal liver metastasis	Intervention	20	61 (56–66)*	65	NR	29.2 ± 4.1
		Control	17	62 (53–72)*	77	NR	29.3 ± 4.2

Age and BMI values are presented as mean ± standard deviation, unless otherwise noted

*Range reported, rather than standard deviation

*NACRT* neoadjuvant chemoradiotherapy, *NR* not reported, *BMI* body mass index, *ASA* American Society of Anesthesiologist class

All studies included participants that were scheduled to undergo surgical resection for gastrointestinal cancer, including colorectal cancer (*n* = 6), gastric cancer (*n* = 1), colorectal liver metastasis (*n* = 1), and hepatocellular carcinoma (*n* = 1) [23–31]. In addition to patients with malignant disease, two studies included patients undergoing colorectal resection for benign disease [23, 29]. These studies included patients with benign colorectal lesions, such as diverticulitis and ulcerative colitis, or patients undergoing colonic reconstruction of non-active inflammatory bowel disease.

### Interventions

The PET programs are summarized in Table 2. All interventions involved aerobic training but varied in terms of activities, frequency, duration, and intensity. Two studies allowed subjects to choose their desired form of aerobic exercise, while the other studies prescribed walking, cycling, or use of other aerobic exercise machines. Only two studies prescribed aerobic exercise alone [29, 31]. In six studies, additional exercises were included, such as resistance training (*n* = 5) [23–25, 27, 30], stretching (*n* = 3) [24, 28, 29], inspiratory muscle training (*n* = 1) [25], or a warm-up and cool-down (*n* = 5) [25, 27–29, 31]. Interventions were solely clinic-based (*n* = 2) [26, 31], solely home-based (*n* = 6) [23–25, 27, 29, 30], or partially clinic-based and home-based (*n* = 1) [28].

**Table 2:** Summary of preoperative exercise therapy regimens

Study	Groups	Length of therapy	Frequency	Duration	Intensity	Follow-up	Outcome
Carli et al.	Biking/strengthening	59.0 days*	Daily	20–45 min	≥ 50% MHR. Volitional fatigue within 8–12 repetitions.	2–4 m	↔Functional walking capacity before surgery ↑ Functional walking capacity in controls 4 weeks after surgery ↔ Length of stay ↔ Postoperative complications ↔ QOL
	Walk/breathing	45.4 days*	Daily	40–45 min		2–4 m	
Cho et al.	Aerobic/resistance training, stretching	4 weeks	3–7/week	NR	NR	1 year	↓ BMI, Body weight, abdominal circumference, volume of visceral fat ↓Postoperative complications ↓ Length of stay
	Matched controls in database	NA	NA	NA	NA	NA	
Dronkers et al.	Aerobic exercise, resistance training, IMT	2–4 weeks	Daily	30–60 min	55–75% MHR, 11–13 on Borg Scale	NR	↑ Respiratory muscle endurance in preoperative period ↔ Time-up-and-go, chair rise time, LASA Physical Activity Questionnaire, physical work capacity, and quality of life ↔ Postoperative complications ↔ Length of stay ↔ QOL
	Home-based exercise advice	2–4 weeks	Daily	≥ 30 mins	NR	NR	
Gillis et al.	Aerobic/resistance training, nutritional therapy, relaxation exercises before and after surgery	4 weeks pre-op, 8 weeks post-op	≥ 3/week	50 mins	≥ 40% HRR, 8–12 repetitions	8 weeks	↑ Functional walking capacity before surgery, and at 8 weeks post-op ↑ Recovery to baseline ↔ Postoperative Complications ↔ Length of stay ↔ QOL
	Aerobic/resistance training, nutritional therapy, relaxation exercises after surgery	8 weeks post-op	≥ 3/week	50 mins	≥ 40% HRR, 8–12 repetitions	8 weeks	
Kaibori et al.	Aerobic exercise, diet therapy	≤ 1 month pre-op, 6 months post-op	3/week	60 mins	NR	6 m	↓Body mass, fat mass ↓Serum insulin, insulin resistance ↑AT VO_2_, peak VO_2_
	Diet therapy	≤ 1 month pre-op, 6 months post-op	NA	NA	NA	6 m	
Kim et al.	Aerobic exercise	4 weeks	Daily	20–30 min	40–65% HRR	4 weeks	↓Heart rate, oxygen consumption ↑Peak power output ↔Functional walking capacity
	Standard care	NA	NA	NA	NA	4 weeks	
Li et al.	Aerobic/resistance training, diet therapy, relaxation exercises	21–46 days	3/week	30 mins	50% MHR; volitional fatigue	8 weeks	↑Functional walking capacity at 4 weeks and 8 weeks postoperatively ↑ Recovery to baseline ↔Postoperative complications ↔ Length of stay ↑ QOL
	Standard care	NA	NA	NA	NA	8 weeks	
West et al.	Aerobic exercise	6 weeks	3/week	40 mins	Intervals at 80% pVO_2_ work rate at θ_L_ and 50% of the difference between in work rates between pVO_2_ and VO_2_ at θ_L_	6 weeks	↓ VO_2_ at θ_L_ after NACRT ↑ VO_2_ at θ_L_ after exercise
Dunne et al.	Aerobic exercise	4 weeks	12 sessions	30 mins	Interval training alternating at less than 60% pVO_2_ at peak and more than 90% pVO_2_ at peak	4 weeks	↑ VO_2_ at AT and peak after exercise ↑ Mental and overall QOL
	Standard care	NA	NA	NA	NA	4 weeks	

Length of therapy refers to therapy prior to surgery, unless otherwise noted

*Mean: ↑ = statistically significant increase compared with control group, ↓ = statistically significant decrease compared with control group, ↔ = no significant difference between groups

*AT* anabolic threshold, *HRR* heart rate reserve, *IMT* inspiratory muscle training, *MHR* maximal heart rate, *NACRT* neoadjuvant chemoradiotherapy, *NA* not applicable, *NR* not reported, *QOL* quality of life, *VO*_*2*_ oxygen uptake, *θ*_*L*_ lactate threshold

### Frequency

In each of the nine studies, the length of the PET program ranged from 2 to 6 weeks. Two studies required participants to resume the exercise program postoperatively and to continue therapy for 8 weeks [27] or 6 months [28]. The frequency of exercise per week varied from daily (*n* = 3) to at least 3 days per week (*n* = 6).

### Duration

In total, each exercise session lasted between 20 and 60 min. The prescribed duration of each session was constant in seven studies [24–28, 30, 31], whereas the duration was incrementally increased over the course of the intervention period in two studies [23, 29]. Resistance training accounted for approximately 10 to 20 min of each exercise session [23, 27] or the time required to reach volitional fatigue [30].

### Intensity

The prescribed aerobic exercise program was customized to each patient in order to achieve the desired intensity and varied between studies. Five studies required subjects to perform aerobic exercise at ≥ 40% of heart rate reserve, at ≥ 50% of maximal heart rate, and/or at a perceived exertion of 11 to 16 on the Borg Scale [23, 27, 29, 30, 32]. In three of these studies, aerobic intensity was incrementally increased over the PET period [27, 29, 31]. One study varied from moderate- to high-intensity exercise [26].

### Control groups

Five studies provided no exercise regimen or other interventions to their control groups [24, 26, 29–31]. In the remaining studies, controls received home-based exercise advice (*n* = 3) [23, 25, 27], diet therapy (*n* = 2) [27, 28], and/or anxiety-reduction techniques (*n* = 1) [27]. Two control groups were also provided with diet therapy or anxiety-reduction techniques (*n* = 2) [27, 28]. These therapies were also offered to their respective intervention groups, in addition to aerobic exercise.

### Compliance

Of the six studies reporting compliance, adherence to the PET program ranged from 16 to 97% [23, 25, 26, 29–31]. Three studies were completed partially or completely in a clinical setting, and compliance was assessed by attendance at those sessions [25, 26, 31]. Kim et al. assessed compliance through a diary, as well as visits to the home by a physical therapist. [29] Similarly, Li et al. assessed compliance through weekly phone calls [30]. In the study by Carli et al., a member of the research team visited the home at least once to verify compliance and telephoned the patient weekly until surgery [23].

### Outcomes

Study outcomes are summarized in Table 2. Heart rate, peak power output, and pVO_2_ were the most responsive measures of physical fitness after PET [29]. West et al. reported that VO_2_ at lactate threshold increased on average by 2.1 ml kg^−1^ min^−1^ after 6 weeks of exercise (*p* < 0.001) [31]. Similarly, two other studies found that both the anaerobic threshold VO_2_ and pVO_2_ were significantly increased in the exercise group [26, 28]. Kim et al. reported that oxygen uptake at a submaximal workload decreased by 13 ± 15% (*p* < 0.05) [29]. Functional walking capacity, as measured by a 6-min walking test, was also examined in several studies and demonstrated mixed results [23, 27, 29, 30].

Postoperative outcomes were reported in several studies. Of the five studies that recorded length of stay, only Cho et al. found that PET significantly reduced this metric (9.0 vs. 10.0 days; *p* = 0.038) [24]. Likewise, of the six studies that examined postoperative complications, only Cho et al. reported that intra-abdominal complications were significantly reduced among the subjects in the exercise group (OR 0.12, 95% CI 0.00–0.89, *p* = 0.033) [24].

Three studies found no differences in quality of life between the intervention and control groups, as assessed through the 36-Item Short Form Survey from the RAND Medical Outcomes Study (SF-36) [27], European Organisation for Research and Treatment of Cancer (EORTC) Quality of Life Questionnaire [25], and Hospital Anxiety and Depression Scale (HADS) [23, 27]. Two studies found significant increases in scores in the general health and mental health components of the Short Form-36 in the PET group (*p* < 0.05) [26, 30].

## Discussion

This systematic review summarizes the recent literature on whole-body PET prior to surgery for patients with gastrointestinal cancer, with the aim of identifying the optimal strategy for decreasing perioperative risks associated with major abdominal surgery. Nine relevant studies, which included 534 patients, were identified. Overall, the articles provided evidence on the clinical benefits of PET in terms of physical fitness, anthropometrics, metabolism, and recovery.

In this systematic review, it was found that PET programs were effective in decreasing heart rate and oxygen consumption and in increasing peak power output in surgical patients with gastrointestinal cancer. Functional walking capacity was the most commonly reported measure of physical fitness and demonstrated mixed results following PET [23, 27, 29, 30]. Such metrics were likely chosen due to the ease of data collection, objectivity, and reliability in the assessment of functional exercise capacity [33, 34]. Additional measurements of physical fitness, including BMI, body weight, abdominal circumference, and fasting serum insulin, were reported in two studies and found to be significantly improved after PET [24, 28].

It is well known that exercise improves physical fitness, specifically cardiopulmonary function, muscle strength, bone mineral density, body weight, adipose tissue mass, and fatigue [35]. However, the effect of exercise on physiological function in cancer patients undergoing GI surgery has not been well elucidated. Several studies have reported the effect of exercise on physical fitness in breast cancer patients and survivors. In one systematic review of breast cancer patients undergoing aerobic or resistance exercise therapy, Markes et al. reported that exercise improved cardiorespiratory fitness, reduced fatigue, and decreased weight [36]. Similarly, McNeely et al. demonstrated that exercise led to increased physical functioning, improved peak oxygen consumption, and decreased fatigue, in a review study of breast cancer patients and survivors [37].

The current review indicates that patients who underwent PET were more likely to recover to their baseline functional capacity after surgical resection [27, 30]. Additionally, in a re-analysis of the data presented by Carli et al. [23], it was demonstrated that postoperative recovery to baseline in the intervention group was most likely to occur in patients whose physical function improved during the PET period, as compared to those whose fitness decreased or remained the same [38].

In this review, the effect of PET on length of stay and postoperative complications demonstrated varying results. Six studies reported these outcomes, but only Cho et al. found a significant decrease in length of stay and intra-abdominal complications. Existing literature reports that exercise improves the postoperative course in other patient populations. For example, in a systematic review by Hulzebos et al., preoperative physical therapy in elective cardiac surgery patients was found to be associated with significantly decreased postoperative pulmonary complications and length of stay [39]. These promising results indicate an urgent gap in, and need for, large-scale, randomized controlled trials in the surgical oncology population.

Five studies evaluated quality of life after PET and provided conflicting results. In the present study, two studies reported a significant improvement in quality of life after PET, with increased scores found in the general health and mental health components of the Short Form-36 [26, 30]. In general, newly diagnosed cancer patients experience a significant reduction in quality of life, with fatigue and emotional distress contributing most to this status [40]. For this reason, exercise has been proposed as a means to enhance cardiorespiratory fitness, lessen fatigue, and improve quality of life in cancer patients [41]. In a study by Courneya et al., quality of life was significantly improved in colorectal cancer survivors who increased their cardiovascular fitness [42].

In this review, three studies included aerobic exercise alone [26, 29, 31], while the others incorporated resistance training [23–25, 27, 30], stretching [24, 25, 28], or inspiratory muscle training into the PET program [25]. In combination with aerobic exercise, these additional interventions may provide further benefits. For example, resistance training has demonstrated effectiveness in reversing sarcopenia in elderly individuals [43–45], and preoperative inspiratory muscle training has shown benefits in the reduction of postoperative pulmonary complications in patients undergoing cardiac surgery [46].

Optimal duration of exercise intervention prior to surgical intervention was not clearly addressed in the studies reviewed. With the shift towards preoperative chemotherapy and radiation for gastrointestinal cancer patients, we may have more time available to optimize patients or preserve function. This will undoubtedly lower the barrier to implementing preoperative exercise interventions of gastrointestinal cancer patients. Future studies will need to focus on not only optimal duration of intervention but also timing of exercise intervention during the preoperative phase.

This review has several limitations. First, there was significant heterogeneity between studies, in terms of design, participants, and interventions, which prevented meta-analysis. The second limitation is that several studies contained a high risk of bias. Two studies had compliance rates less than 50%, while three studies did not report the compliance rate. Third, given the limited number of studies, we were not able to examine specific cancer subtypes or specific populations including the elderly, frail, those with morbid obesity, or those with significant comorbidities. Finally, this review did not exclude studies based on surgical technique. This may have confounded some results, as laparoscopy is associated with short length of stay and decreased postoperative morbidity. Despite these limitations, this review provides a comprehensive assessment of preoperative exercise regimens developed for patients with gastrointestinal malignancies.

## Conclusions

Preoperative exercise therapy is associated with improved physical fitness and recovery to baseline functional capacity in patients undergoing surgical resection of gastrointestinal cancer. The effect of PET on length of stay, postoperative complications, and quality of life in the surgical patient with gastrointestinal cancer remains unclear. There is an urgent need for large-scale, randomized controlled trials that examine the optimal duration, frequency, and intensity of preoperative exercise programs.

## Abbreviations

ASA: American Society of Anesthesia
DARE: Database of Abstracts of Reviews of Effectiveness
EORTC: European Organisation for Research and Treatment of Cancer
HADS: Hospital Anxiety and Depression Scale
ICTRP: International Clinical Trials Registry Platform
OR: Odds ratio
PET: Preoperative exercise therapy
PRISMA: Preferred Reporting Items for Systematic Review and Meta-Analysis
PROSPERO: Prospective Register of Systemic Reviews
